# Synergistic computational and experimental studies of a phosphoglycosyl transferase membrane/ligand ensemble

**DOI:** 10.1016/j.jbc.2023.105194

**Published:** 2023-08-25

**Authors:** Ayan Majumder, Nemanja Vuksanovic, Leah C. Ray, Hannah M. Bernstein, Karen N. Allen, Barbara Imperiali, John E. Straub

**Affiliations:** 1 Department of Chemistry, Boston University, Boston, Massachusetts, USA; 2 Program in Biomolecular Pharmacology, Boston University School of Medicine, Boston, Massachusetts, USA; 3 Department of Biology, Massachusetts Institute of Technology, Cambridge, Massachusetts, USA; 4 Department of Chemistry, Massachusetts Institute of Technology, Cambridge, Massachusetts, USA

**Keywords:** polyprenol phosphate, glycoconjugate biosynthesis, membrane protein, molecular dynamics, protein dynamics

## Abstract

Complex glycans serve essential functions in all living systems. Many of these intricate and byzantine biomolecules are assembled employing biosynthetic pathways wherein the constituent enzymes are membrane-associated. A signature feature of the stepwise assembly processes is the essentiality of unusual linear long-chain polyprenol phosphate-linked substrates of specific isoprene unit geometry, such as undecaprenol phosphate (UndP) in bacteria. How these enzymes and substrates interact within a lipid bilayer needs further investigation. Here, we focus on a small enzyme, PglC from *Campylobacter*, structurally characterized for the first time in 2018 as a detergent-solubilized construct. PglC is a monotopic phosphoglycosyl transferase that embodies the functional core structure of the entire enzyme superfamily and catalyzes the first membrane-committed step in a glycoprotein assembly pathway. The size of the enzyme is significant as it enables high-level computation and relatively facile, for a membrane protein, experimental analysis. Our ensemble computational and experimental results provided a high-level view of the membrane-embedded PglC/UndP complex. The findings suggested that it is advantageous for the polyprenol phosphate to adopt a conformation in the same leaflet where the monotopic membrane protein resides as opposed to additionally disrupting the opposing leaflet of the bilayer. Further, the analysis showed that electrostatic steering acts as a major driving force contributing to the recognition and binding of both UndP and the soluble nucleotide sugar substrate. Iterative computational and experimental mutagenesis support a specific interaction of UndP with phosphoglycosyl transferase cationic residues and suggest a role for critical conformational transitions in substrate binding and specificity.

Phosphoglycosyl transferases (PGTs) catalyze the transfer of a phosphosugar group from a UDP-sugar substrate to a membrane-resident polyprenol phosphate (most commonly in bacteria undecaprenol or decaprenol phosphate) acceptor (1). This reaction is the initiating step of many glycoconjugate biosynthesis pathways (2, 3, 4). The current studies focus on the monotopic PGT (monoPGT) superfamily (3, 5). The first structure for a monoPGT superfamily member, the *Campylobacter concisus* PglC (Protein Data Bank [PDB] 5W7L), was determined in 2018 (6). This structure (6) and extensive sequence analyses (5, 7) show that all superfamily members include a minimal catalytic domain defined by a reentrant membrane helix (RMH) and an active site including an Asp-Glu catalytic dyad and magnesium cofactor positioned at the membrane interface. Subsequent computational analyses were also applied to define the sequence motifs that guide the RMH topology (8). Catalysis proceeds *via* a covalent intermediate (9) with both soluble and amphiphilic substrates remaining in aqueous and membrane environments, respectively (10). Although the novel structure provided new insights, the enzyme was crystallized in a detergent-solubilized form and lacked the native context of the membrane. Indeed, the snapshot of the structure that was captured represented an open conformation with direct access of bulk solvent to the active site. The suggestion of a catalytically competent closed conformer, which could lead to a protected active site, came from bioinformatic covariance analysis of PGT orthologs. The use of covarying residues to indicate structural contacts pointed to the interaction of the RMH with the catalytic core of the protein as well as the interaction of structural elements that would allow active site closure (5, 7). *In vivo* analysis using the substituted cysteine accessibility method (method (11) and the observation of an ordered phosphatidylethanolamine head group in the crystal structure corroborated the monotopic topology and the placement of key membrane-interacting residues in PglC (6, 8). Additional information on the monoPGT and its membrane-bound substrate was also derived from studies using the model membrane styrene maleic acid lipoparticle method (12, 13). These studies showed that the conserved UndP, which features a signature arrangement of *E* and *Z* isoprene units, in contrast to the all-*E* solanesol phosphate is selectively recruited to the PGT enzyme (14).

Despite the progress in understanding this first committed step in glycoconjugate biosynthesis, there is still a gap in our understanding of the dynamic interactions between the membrane-bound PGT and its membrane-associated UndP substrates in a native lipid bilayer environment. Our snapshot views from structural analysis, employing X-ray crystallography, provided insight into membrane association; however, the analyses with detergent-solubilized protein, would have disrupted native membrane interactions and precluded assessment of the contribution of the membrane environment to the subtle interplay of protein dynamics and ligand interactions that lead to function. The analysis of membrane-bound enzymes in their native environment has revealed unexpected properties that impact catalysis, as illustrated for example in the dramatic enhancement of catalysis observed for rhomboid proteases in membrane (15). A recent study utilizing native mass spectrometry in concert with molecular dynamic simulations showed that the lipid I biosynthetic membrane enzyme MraY exists in a monomer-dimer equilibrium where dimerization is favored by binding of the lipid substrate undecaprenyl phosphate as well as the product lipid I (16).

Major opportunities in the study of membrane proteins in native-like environments have emerged from the development of liponanoparticles such as nanodiscs, stabilized by membrane scaffold proteins, introduced by Sligar and styrene maleic acid lipoparticles, stabilized by amphiphilic copolymers such as styrene maleic acid (reviewed in (17, 18)). Indeed, purified and stabilized samples of PglC using such membrane mimetic systems have been reported (13, 19). However, although these systems are very attractive for some studies certain limitations must be recognized. For example, the area of the membrane is limited (10–20 nm diameter), the boundaries between the membrane and the liponanoparticle scaffold are nonnative and account for much of the available area, and additives such as divalent cations can be destabilizing. Importantly, the precision/resolution of experimental distance measurements such as FRET may be inadequate for the questions at hand.

Herein, we present a synergistic computational and experimental approach for analysis of dynamics of the monotopic PGT, PglC, and interactions with both the membrane and the unique membrane-bound substrate undecaprenol phosphate (Fig. 1). We apply the CHARMM36 force field, which is the most widely used all-atom resolution force field for lipid systems. This force field has been used extensively in simulations of eukaryotic, prokaryotic, and artificial membranes. Specifically, we validate a model representing the experimentally determined catalytic core of PglC (ca. 200 residues) and a lipid bilayer (400 phospholipids/200 per leaflet) of relevant composition and dimensions, with and without the UndP. These studies show that the UndP adopts a compact conformation in a single leaflet of the bilayer; the inclusion of PglC shows that the UndP conformation mirrors the position of the reentrant helix making critical interactions with cationic residues. Molecular dynamics reveals motions that would promote a closed active site which is also supported by the observation of conformers in an experimental crystal structure.

**Figure 1:**
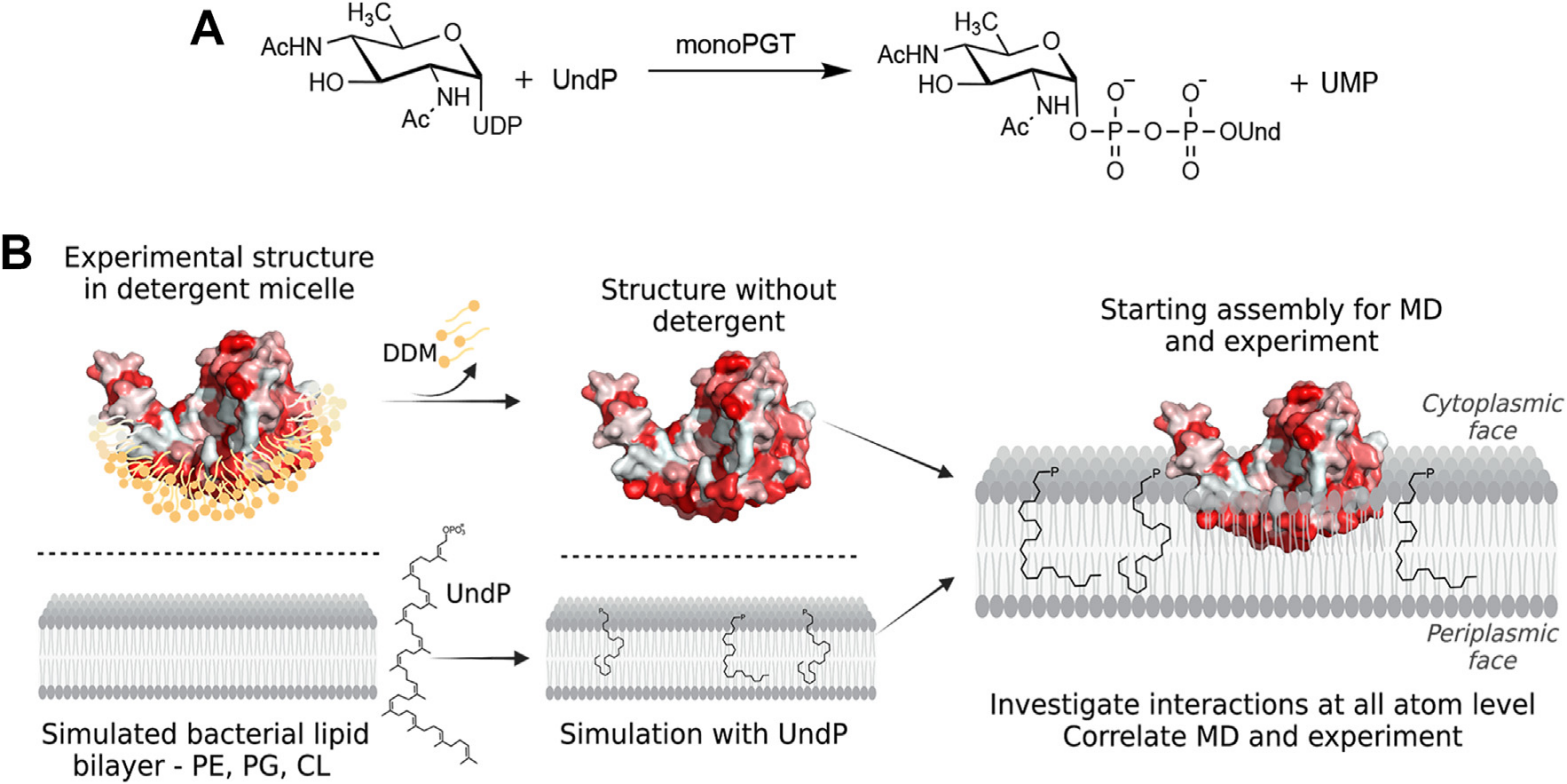
**MonoPGT reaction and overview of goals.***A*, phosphoglycosyl transferase reaction between soluble cytosolic UDP-diNAc-bacillosamine (Bac) and membrane-associated UndP to afford membrane embedded UndPP-Bac and UMP. *B*, schematic representation of experimental design with panel showing initial assembly for MD simulations. The surface is color ramped as hydrophobic (*red*) to hydrophilic (*white*) using the Eisenberg hydrophobicity scale (see Experimental procedures). MD, molecular dynamics; UndP, undecaprenol phosphate.

## Results and discussion

### Simulated bacterial membrane exhibits a characteristic liquid-disordered phase

We first simulated a number of lipid bilayers without protein, to validate the membrane model against experimental observables including area per lipid. As a benchmark, we simulated a lipid bilayer composed of 67 mol% 1-hexadecanoyl-2-(9*Z*-octadecenoyl)-*sn*-glycero-3-phosphoethanolamine (POPE), 23 mol% 1-hexadecanoyl-2-(9*Z*-octadecenoyl)-*sn*-glycero-3-phosphoglycerol (POPG), and 10 mol% cardiolipin (CL) of defined acyl chain composition (Fig. S1) to mimic the experimentally determined average composition for typical inner membranes of Gram-negative bacteria including *C. concisus* (20). A previous experimental study (14) employed pyrene as a membrane probe to assess changes in membrane polarity in the presence of UndP. Only modest effects on the membrane structure were observed on inclusion of 0.5 mol% UndP. In contrast, another study predicted the formation of a nonlamellar hexagonal phase in membrane containing 5 mol% UndP (21). The focus of this work is on physiologically reasonable concentrations of 0.5 to 1.0 mol% UndP where a lamellar bilayer phase is expected. To assess computationally the degree of lipid order and packing in the absence and presence of UndP, the liquid crystal order parameter (P_2_) and 2D bond-orientational order parameter (| $\Psi_{6}^{k}$ |) were computed for all lipid molecules. The P_2_ order parameter has been used to understand the organization of lipid tails in membrane domains, in both coarse-grained and all-atom models (22, 23). The value of the P_2_ order parameter reports on the orientation of the lipid director vector relative to the vector normal to the membrane. The range of P_2_ values varies from perpendicular (−0.50), to parallel (1.0). The P_2_ value for a liquid ordered domain of a lipid bilayer is greater than 0.9, using the CHARMM36 force field (22). In contrast, studies using the coarse-grained MARTINI model have reported P_2_ values varying from 0.63, for liquid disordered domains, to 0.82, for liquid ordered domains (23). The absolute value of the 2D bond-orientational order parameter ${|\Psi }_{6}^{k}|$ estimates the extent of hexagonal packing around the lipid. A high degree of hexagonal packing is a hallmark of the liquid ordered domain. The ${|\Psi }_{6}^{k}|$ value varies from 0.42, for liquid disordered domains, to 0.48, for liquid ordered domains (23). The values of the P_2_ and ${|\Psi }_{6}^{k}|$ order parameters calculated from our simulations are tabulated in Table 1. The distribution of the order parameters obtained from the simulation is shown in Fig. S2.

**Table 1:** Values of P_2_ and ${|\Psi }_{6}^{k}|$ order parameters of different membrane components, measuring nematic order and hexagonal packing, respectively, obtained from simulation of membrane with and without the inclusion of UndP

Order parameters	System	POPE	POPG	CL
P_2_ value	membrane	0.67 ± 0.02	0.69 ± 0.03	0.71 ± 0.04
	membrane with UndP	0.67 ± 0.02	0.69 ± 0.04	0.72 ± 0.04
${|\Psi }_{6}^{k}|$ value	membrane	0.40 ± 0.02	0.41 ± 0.03	0.41 ± 0.02
	membrane with UndP	0.40 ± 0.02	0.41 ± 0.03	0.40 ± 0.02

In comparison with previous studies, the lower values of the P_2_ and ${|\Psi }_{6}^{k}|$ order parameters indicate that the lipid bilayer in this composition forms a liquid-disordered phase at room temperature. The distribution of the ${|\Psi }_{6}^{k}|$ order parameter over different regions of the membrane shows no phase separation or domain formation in either leaflet (Fig. S3). Parallel studies without CL showed that the major order parameters of the membrane components were not significantly perturbed (Fig. S4).

### Undecaprenol phosphate (UndP) is disordered and localized in a single leaflet

Linear polyprenol phosphates (PrenPs) play a critical role in glycoconjugate biosynthesis and are present in concentrations estimated to be <0.1 mol% in bacterial membranes (19, 24). The structures of PrenPs have been explored through computational and experimental studies (21, 25, 26). Previous computational studies showed that dolichol (C95), dolichol phosphate (C95-P), and UndP feature three domains characterized by a central coiled region, involving a series of *Z*-configuration isoprene units, and two flanking regions, including the polar head group and the tail with *E*-configuration isoprene units (26). In a study of C95 and C95-P in a dimyristoylphosphatidylcholine bilayer, the polar head groups of the PrenP were observed to be located near the membrane-water interface with the polyprenyl coiled region interacting with the phospholipid acyl tails.

**Figure 2:**
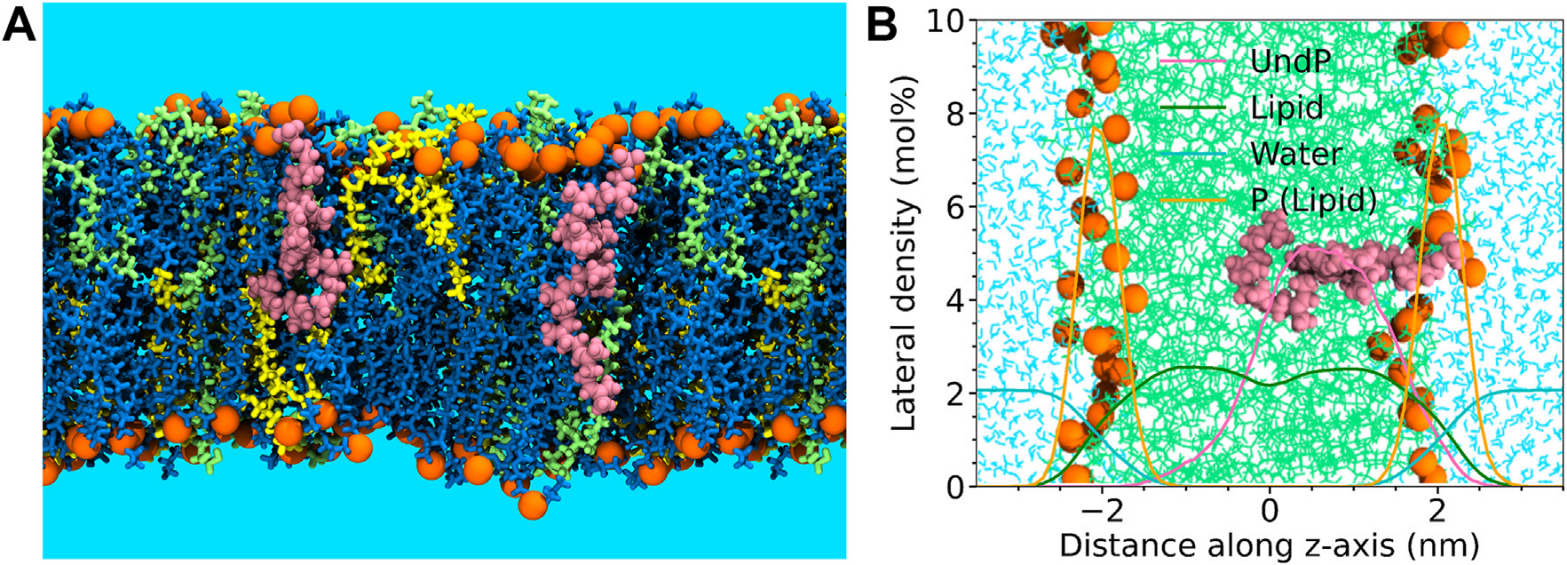
**UndP spans a single leaflet using phosphate group as an anchor at the lipid-water interface.***A*, pictorial representation of the lipid bilayer composed of POPE (*blue*), POPG (*green*), CL (*yellow*), and UndP (*salmon*). Lipid head groups are represented in *orange*. *B*, density distribution of different membrane components. Traces represent the density distributions of UndP (*salmon*), lipid acyl chains (*green*), water (*cyan*), and phospholipid phosphate head group (*orange*), respectively. CL, cardiolipin; POPE, 1-hexadecanoyl-2-(9*Z*-octadecenoyl)-*sn*-glycero-3-phosphoethanolamine; POPG, 1-hexadecanoyl-2-(9*Z*-octadecenoyl)-*sn*-glycero-3-phosphoglycerol; UndP, undecaprenol phosphate.

To investigate the behavior of UndP in this system, we placed two UndP molecules (1.0 mol%) in the lipid bilayer in an extended transmembrane conformation (Fig. 2*A*). The order parameters (Table 1) show that the inclusion of UndP does not perturb the lipid packing. This result is consistent with previous published studies employing pyrene as a probe to assess changes in membrane polarity, which showed only modest effects on the membrane up to 0.5% UndP (14). From the density analysis, the phosphoryl groups of the UndP molecules are observed to be primarily located near the membrane-water interface (Fig. 2*B*). This density profile suggests that the UndP molecule is localized in a single leaflet of the lipid bilayer, in agreement with previous studies (26). We performed Voronoi tessellations by considering one atom per lipid tail to compute the area occupied by the different bilayer components (Fig. S5). The computed areas occupied by POPE, POPG, CL, and UndP were found to be 58.3, 57.7, 108.8, and 31.7 Å^2^, respectively. The average value of the radius of gyration of UndP, calculated over the course of the simulation (Fig. S5), was found to be 0.85 nm. Substantial fluctuations in the radius of gyration of UndP result from the flexibility of the polyprenyl group which readily and frequently undergoes transitions between stretched, coiled, and unstructured conformations.

### Validation of computational model of PglC in a model bacterial membrane

**Figure 3:**
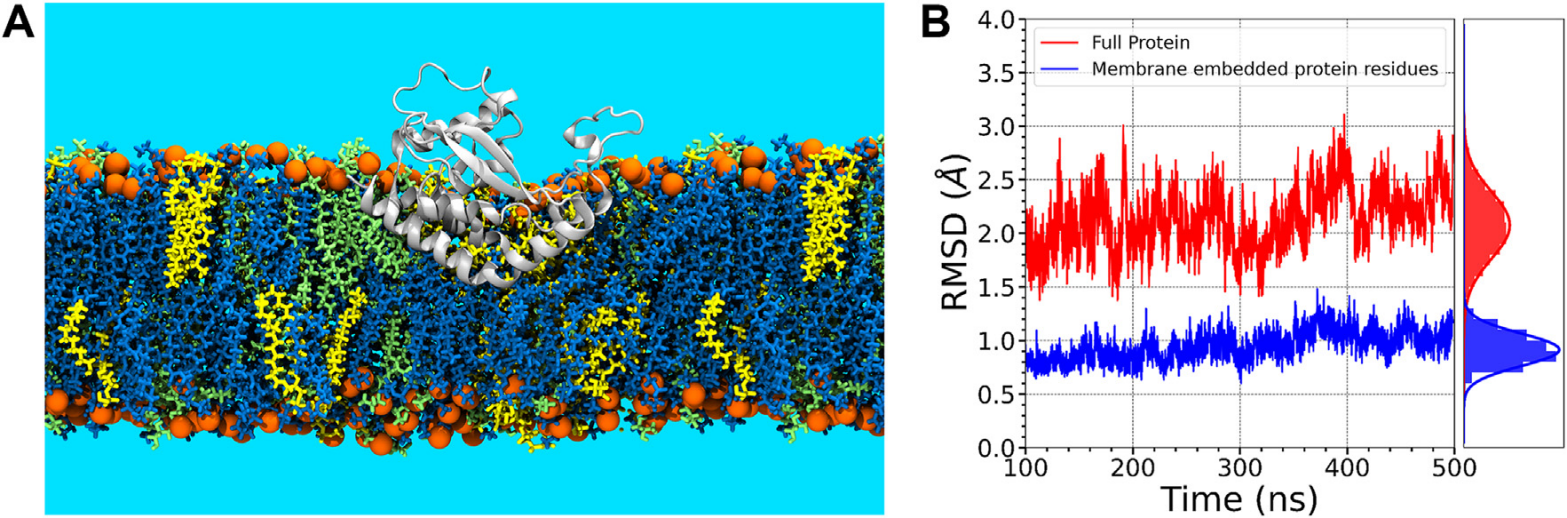
**Simulated structural ensemble shows excellent agreement with experimentally-derived structures.***A*, pictorial representation of the POPE:POPG:CL 67:23:10 lipid bilayer, showing POPE (*blue*), POPG (*green*), and CL (*yellow*). The PglC protein and lipid head groups are represented by *gray*, and *orange*, respectively. *B*, RMSD of PglC structures obtained from simulation along with the cumulative distributions. The crystal structure was used as the reference state. CL, cardiolipin; POPE, 1-hexadecanoyl-2-(9*Z*-octadecenoyl)-*sn*-glycero-3-phosphoethanolamine; POPG, 1-hexadecanoyl-2-(9*Z*-octadecenoyl)-*sn*-glycero-3-phosphoglycerol; UndP, undecaprenol phosphate.

Having validated the membrane model in the absence and presence of UndP, we introduced the PglC protein employing the standard method for protein insertion using the CHARMM-GUI (27). The resultant placement was consistent with previous experimental and computational studies (6, 8) (Fig. 3*A*). The experimentally determined structure of PglC (PDB 5W7L) (6) was used to examine dynamics in the membrane environment (composed of 67 mol% POPE, 23 mol% POPG, and 10 mol% CL, as in the simulations above). We note the C-terminal residues (183–205) were not ordered in the X-ray crystal structure and therefore were excluded from the simulations in all the presented studies.

The RMSD values of the PglC structure over the time course, after the initial computational equilibration, with reference to the experimental structure, is shown in Figure 3*B*. The insertion depth of the protein obtained from the simulation is shown in Fig. S6. The comparison of root mean square fluctuation of PglC with the experimentally derived B-factors (Fig. S7) shows excellent agreement between experiment and simulation. The X-ray crystallographic structure shows that the signature RMH is kinked at the Ser23-Pro24 motif, with a hinge angle of 118^°^. The hinge angle is defined by the angle between two α-helices of the RMH on either side of Ser23-Pro24. In the simulation, the structure of the reentrant membrane helix (residue 1–36) is similar to that in the crystal structure with an RMSD of <1 Å. Those regions of PglC exposed to water show the largest structural fluctuations, although the average RMSD values over the simulation are less than 3 Å. The insertion depth of PglC observed in the simulation is associated with an RMH having a hinge angle, centered about Ser23-Pro24, with an average value of 117^°^. This is consistent with the experimentally observed value of 118^°^. The maximum insertion depth of PglC is 14 Å, also in agreement with structural and biochemical studies (6, 8). These findings validate our computational model of PglC in a model bacterial membrane.

### Role of basic residues and electrostatic steering in UndP binding to PglC

**Figure 4:**
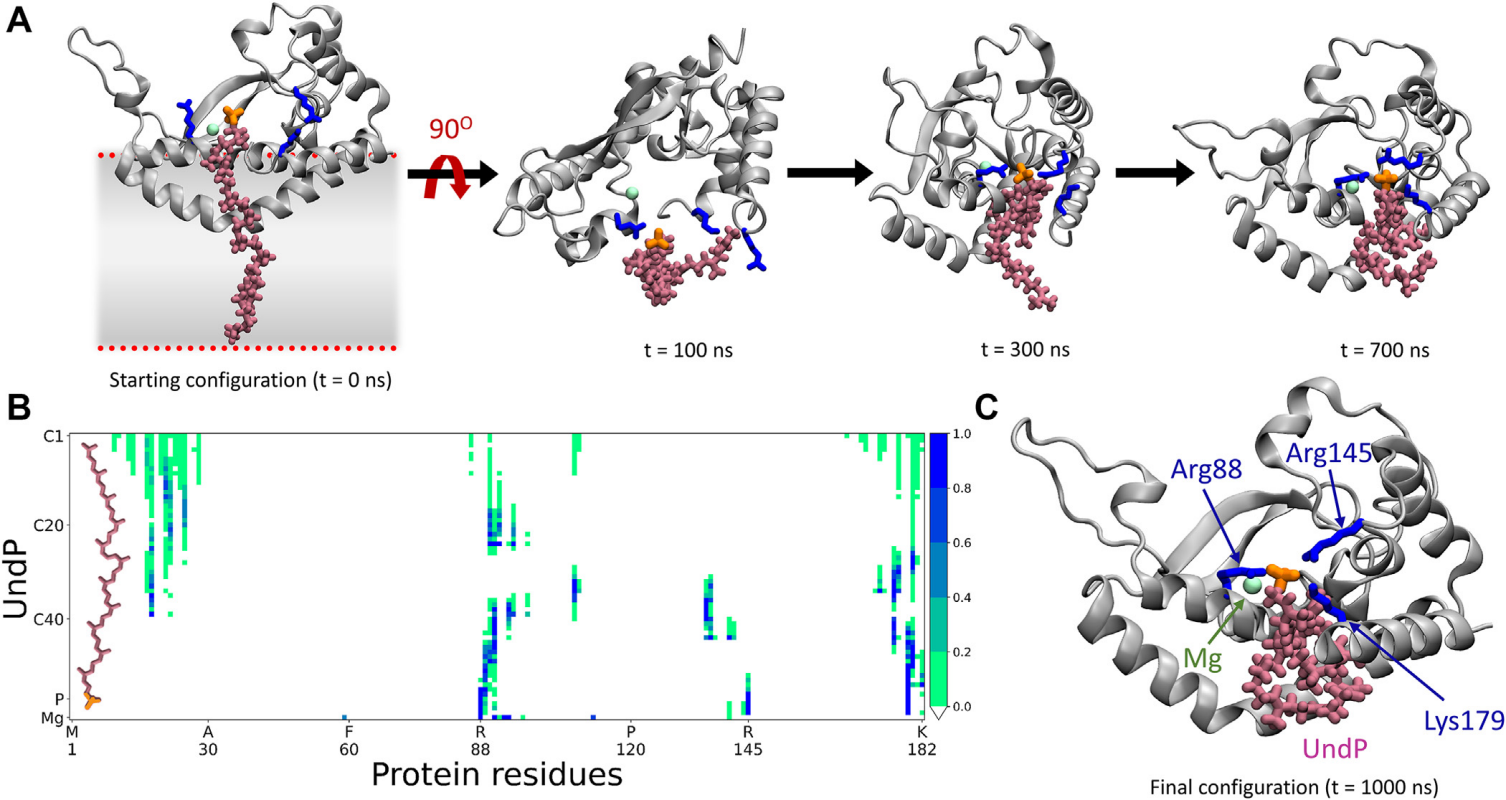
**Basic residues in the PglC active site play an important role in mediating ligand binding.***A*, interaction of PglC and UndP at different times of the simulation. Note the extended, transmembrane starting conformation of UndP. Lipids and water were not shown for clarity. *B*, contact map of UndP and PglC using a 5 Å interaction distance cutoff. The bar colors represent the contact probability, where a value of one represents a contact maintained between the residues throughout the simulation trajectory. *C*, ribbon representation of UndP and PglC interaction. *Gray*, *salmon*, *green*, and *blue* represent the PglC, UndP, Mg^2+^, and residues of PglC that interact with UndP, respectively. The location of the active site and catalytic Asp-Glu motif is coincident with the Mg^2+^ cofactor. UndP, undecaprenol phosphate.

Previously, we reported that the presence of PglC in a model membrane system increases the local UndP concentration by two-fold relative to the bulk membrane (8). It has also been shown that conserved basic residues in the active site cavity of PglC play an important role in the binding of UndP to PglC (7). To identify key interactions between UndP and PglC, we simulated a lipid bilayer using the same composition and parameters as described above, augmented by the inclusion of two UndP molecules (0.5 mol%, see Experimental procedures). This proportion of UndP is somewhat higher than a typical physiological concentration (<0.1 mol%) but is necessary to constrain the size of the system for computation. In the simulation, one UndP molecule was found to interact with the PglC (Fig. 4*A*). The second UndP did not show any specific binding and was observed to diffuse away from PglC over the course of the simulation. The contact map for PglC and the interacting UndP, obtained from simulation, is shown in Figure 4*B*. The resulting binding pose suggests that the interaction of UndP and PglC is mediated by Arg88, Arg145, and Lys179 (Fig. 4*C*).

The computational prediction of Arg88 as a mediator of UndP binding is consistent with a previous mutational analysis (7) showing that the R88Q variant has minimal activity (less than 30% WT activity when tested at a 10-fold higher concentration). In the current studies (Table S1), Arg145 has a large effect when substituted by either alanine or glutamine, with ∼100 to 200-fold reduction in activity when compared to WT for either variant. However, the mutation of Lys179 and Lys182 (singly or in a double mutant) to either alanine or glutamine indicated that these residues do not play a specific role in catalysis; however, we posit that the preponderance of positive charge surrounding the active site may compensate for the replacement of the two lysines.

**Figure 5:**
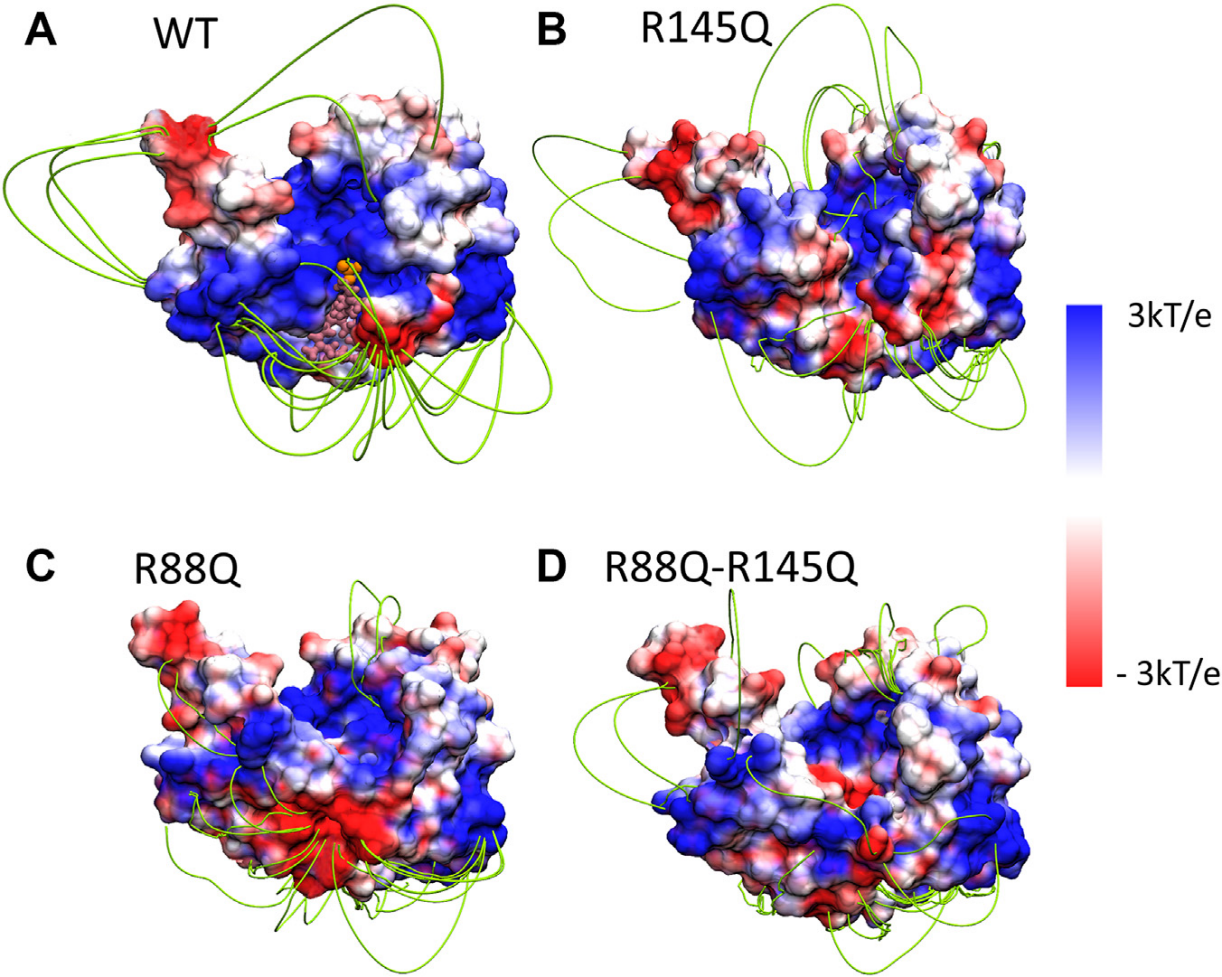
**Electrostatic steering guides the negatively-charged UndP toward the positively-charged PglC active site.** Electrostatic potential map of (*A*) WT, (*B*) R145Q, (*C*) R88Q, and (*D*) R88Q-R145Q PglC. The protein surface color, varying from red to white to blue, represents electrostatic potential values ranging from negative to positive values. The corresponding electric field lines are shown in *green*. UndP is depicted in the active site of WT PglC to indicate the position of the active site. UndP, and the head group of UndP are represented by *salmon*, and *orange* respectively.

Considering these experimental results, we performed parallel simulations of three site-directed variants of PglC, R88Q, R145Q, and R88Q-R145Q, in a membrane with two UndP molecules, as described above. The UndPs were initially positioned as in the WT PglC simulation. In contrast to the simulation of the WT PglC, UndP was not observed to bind to the active site of any variant PglC during a 1 μs simulation. This observation is consistent with the experimental kinetic analysis of the variants; replacement of one or two basic arginine residues with a polar, but neutral, glutamine impacted the binding of the negatively charged UndP. To explore the role of charge in the relative binding affinity of UndP to the WT and variant forms of PglC, we computed the electrostatic potential map (Fig. 5). In the case of WT PglC, the Arg88, Arg145, and Lys179 residues form a patch of positive charge near the active site (Fig. S8). In contrast, the PglC variants have significantly diminished positive charge in the active site region. We postulate that this change in charge distribution disrupts the electrostatic steering of the UndP toward the active site and diminishes the binding affinity. In addition, the R88Q mutation deforms the region encompassing residues 174 to 182 of PglC (Fig. S9). Similar patterns were seen in independent simulations of the R88Q and R88Q-R145Q variants. Along with the diminished positive charge density in the active site, the deformation of this (residues 174–182) region would be likely to negatively impact UndP binding to the active site of PglC.

### Closure of the mobile loop is correlated with occupancy of the active site

**Figure 6:**
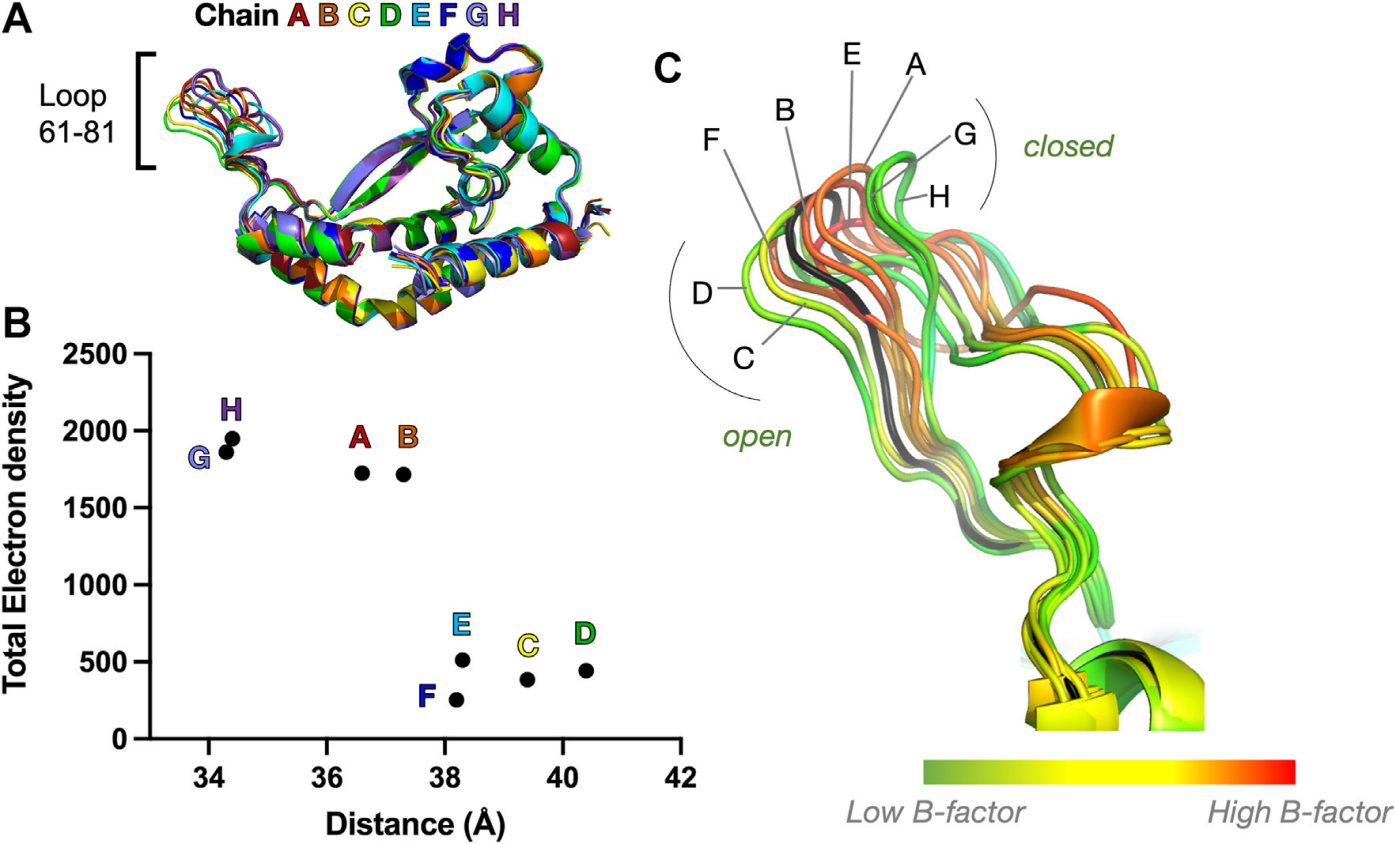
**Mobile loop “closed” conformations observed upon occupancy of PglC active site.***A*, overlay of the eight chains in the WT SeMet dataset. Chains colored by *rainbow* from chain A (*red*) to chain H (*violet*). *B*, total electron density present in each active site from partial occupancy of UDP was calculated as the summation of integrated electron density peaks for each chain using Mapman (see Experimental procedures). The distances were measured from the Cα at the top of the mobile loop (Cα of N70 in all chains except E, where it is Cα A69, due to differences in loop conformation) to the Cα of I121. *C*, B-factors for each residue mapped to the mobile loop. Low B-factors are observed for the “open” and “closed” conformations, correlated with electron density in the active site. For reference, the structure of I57M/Q175M PglC at 2.74 Å resolution (PDB 5W7L) is shown in *black*. PDB, Protein Data Bank.

Closure of the mobile loop (*C. concisus* residues 61–81 Fig. 6), which connects the extended β-strand of the core fold of the monoPGT with the helix proximal to the active site, has been hypothesized to be integral to substrate binding (6). This model is based on two previous observations: (a) the divergence of the loop position between energy-minimized predicted structural models and the crystallographically determined model and (b) conservation of residues within the mobile loop. In the current study, further insight is derived from the structure of selenomethionine (SeMet)-derivatized PglC co-crystallized with UDP. UDP was utilized in co-crystallization, as it significantly stabilized this PGT as shown by nanoscale differential scanning fluorimetry (nanoDSF; Fig. S10). In contrast to I57M/Q175M PglC (6), the SeMet PglC has an asymmetric unit (ASU) comprising eight monomers rather than two per unit cell, allowing independent refinement. Although the resolution was low (3.01 Å), and the ligand was not stoichiometrically bound (10–20% occupancy of any ligand to the active site), it was possible to make correlations between the presence or absence of electron density and the loop conformations. When the eight copies of PglC in the ASU were overlaid, the mobile loop is the main region of structural divergence between the chains (Fig. 6*A* and Table S2). In monomers G and H, which are closed, as well as A and B (intermediate conformation), a comparatively large number of electrons is present in the site where the nucleotide would be expected to bind. In contrast, monomers C, D, E, and F, which are open, all have significantly fewer electrons present (Fig. 6*B*). Analysis of the mobile loop in the context of active-site ligand occupancy shows that chains with a greater positive electron density were in a more closed conformation than those with little or no density. Monomers occupying the same position within each of the four lattice dimers do not always occupy the same mobile loop position; thus, the difference in the observed loop conformation is likely due to differential binding at the active site. Mapping of crystallographic B-factors in each monomer reveals that the loops occupy either one of two relatively low-B-factor conformations corresponding to open (C and D) or closed (G and H), with higher B factor loops occupying positions between these two extremes (A, B, E, and F) (Fig. 6*C*).

### Correlation of mobile loop dynamics in simulation and experiment

**Figure 7:**
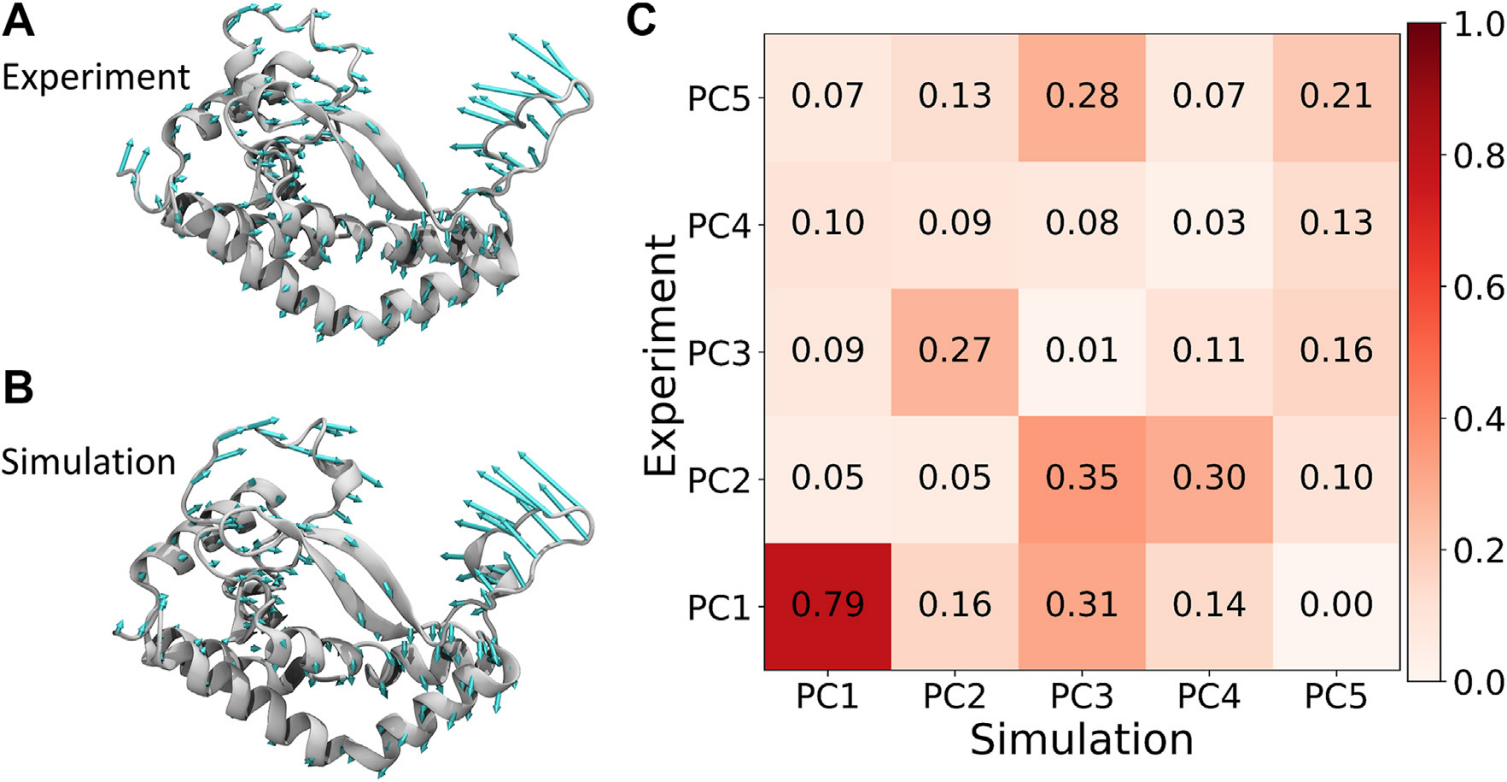
**Similar loop-closing motion observed in computationally- and experimentally-derived structures.** Movement of the protein along first principal component obtained from (*A*) experiment and (*B*) simulation. *C*, inner product of the eigenvectors associated with each PC obtained from experiment and simulation. PC, principal component.

We investigated the loop-closing motion of PglC using two independent means: structures derived from our computer simulations and a series of eight experimentally derived PglC crystal structures. In both cases, we apply principal component analysis to identify essential motions of the protein (28, 29). The resulting principal components (PCs) provide insight into conformational differences of the protein in the crystal environment and observed in molecular dynamics simulations (Fig. 7, *A* and *B*).

In both cases, we found that the loop closing motion corresponds to the eigenvector characterizing the first principal component. To provide a quantitative comparison of the two predicted principal components, we projected one principal component onto the other to determine the quantitative overlap or similarity. To compare the PCs from structures derived from experiment and simulation, we computed the inner product of the first five PCs. The inner product varies from highest (1, identical PCs) to lowest (0, no similarity) degree of overlap (Fig. 7*C*). In each case, the first PC is dominated by motion of the mobile loop of PglC. We observe a high degree of similarity for the first PCs, demonstrating strong correlation between the large-scale loop dynamics observed in simulation, in the membrane environment, and obtained from experiment, in the crystal environment.

Our results qualitatively and quantitatively demonstrate the similarity between the loop motion in experiment and simulation. This synergistic comparison provides insight into the primary functionally important motion in PglC.

## Conclusions

The challenge of how to investigate both dynamics and interactions within a membrane/ligand/protein ensemble was addressed herein using computation augmented by experiment. We have adopted an iterative approach using computation to yield a functionally relevant view of a monoPGT in the membrane and used the results both to interpret and inform experimental design. The all-atom molecular dynamics simulation, using a lipid bilayer composition representative of the inner membrane of Gram-negative bacteria, was observed to form a liquid-disordered phase at room temperature, a property which was maintained upon addition of UndP. In this ensemble, the phosphate head groups of the UndP are found at the membrane-water interface, whereas the prenyl tails were disordered and largely localized within a single leaflet of the model bacterial membrane. Such an arrangement is expected to be enthalpically equivalent to an extended transmembrane conformation but entropically favored. With respect to the arrangement of the *E* and *Z* isoprene units in UndP, we observe that the compact conformation observed by 1000 ns is consistent with the characteristic set of Z-isoprene units and agree with the lack of binding of the all-*E* solanesol phosphate, observed experimentally.

Assessment of the monoPGT in the simulation showed good agreement between the experimentally determined X-ray crystal structure (solved with detergent-solubilized protein) and the equilibrated computational structure in the membrane. The PglC structure, which is substantively embedded in the membrane (24% embedded in contrast to the 3.9% average for monotopic membrane proteins) (6), was found to undergo fluctuations with low RMSD values (<3 Å) in comparison to the crystal structure. The N terminus of the protein forms a reentrant membrane helix by creating a 117^o^ angle at the hinge centered on the Ser23-Pro24 residues. These computationally derived observations of the protein within the lipid bilayer correlated well with observations derived from experiment.

The analysis also provided a high-level view of the membrane embedded PglC/UndP ensemble. The findings further suggested that it is advantageous for the polyprenol phosphate to largely occupy the leaflet where the reentrant membrane helix resides as opposed to additionally disrupting the opposing leaflet of the bilayer, which would be energetically costly. The computational approach yielded the first view of a polyprenol phosphate binding to a monoPGT active site, revealing that the basic residues Arg88 and Arg145 of PglC play a critical role in facilitating the recognition and binding of UndP. This observation highlights a specific mode of interaction of UndP with enzyme and is consistent with experimental results showing that the activity of PglC was significantly reduced when Arg145 or Arg88 were mutated to Ala or Gln. The importance of these residues is underscored by the computed electrostatic potential and associated electric field, which suggests a role for electrostatic steering. Such electrostatic steering likely acts as a major driving force contributing to the recognition and binding of both UndP and the soluble nucleotide sugar substrate. Both substrates must overcome significant energetic barriers in binding to the active site—desolvation in the case of the UDP-sugar and competition with lipid head group interactions in the bulk membrane in the case of UndP.

The application of theory elucidated the molecular basis underlying experimental observations and validated differences in structural conformations as functionally relevant. It was previously conjectured that closure of the mobile loop may play an important role in substrate binding and attainment of the catalytically competent conformation. Our analysis shows excellent agreement between the simulated ensemble of protein structures in membrane and the experimentally observed structures with varying degrees of active site-ligand occupancy in the crystal environment. In both cases, we observe global fluctuation of PglC between open and closed states, characterized by the position of the mobile loop relative to the active site. Taken together, our results provide a holistic view of the critical role of charge and structural transitions in the recognition and binding of the UndP substrate to a monoPGT and highlight the synergistic use of computation and experiment to assess membrane–resident interactions. More generally, in the case of membrane proteins where structure determination is so challenging and biophysical characterization in model membrane systems has limitations—approaches integrating high-level simulations can help to advance understanding of this critical sector of the proteome.

## Experimental procedures

### Computational models and analysis methods

PglC and UndP were simulated in a lipid bilayer composed of 67 mol% POPE, 23 mol% POPG, and 10 mol% CL of defined acyl chain composition (Fig. S1) using the CHARMM36 m all-atom force field (30, 31). A lipid bilayer composed of 200 phospholipid molecules without PglC was prepared. A second lipid bilayer composed of 400 phospholipid molecules containing UndP and PglC (PDB ID 5W7L) (6) was also prepared. Residues 57 and 175 of PglC were mutated to Ile and Gln, respectively, using Chimera (32) as these mutations were present in the experimental protein structural analysis (and constitute the WT protein used in this study). Each lipid bilayer was solvated using the TIP3P water model (33). The concentration of KCl was set at 0.15 M. The UndP-only and UndP-plus-PglC systems were solvated with 52 and 80 water molecules per phospholipid, respectively. The initial structure of UndP was taken to be an extended transmembrane conformation which was found to span a single leaflet using phosphate group as an anchor in the lipid water interface. The PglC molecule was placed in the leaflet containing the head group of UndP in the simulations of PglC in a model membrane. Each bilayer was equilibrated for a minimum of 50 ns of molecular dynamics simulation in the constant pressure and temperature ensemble, using the Nose–Hoover thermostat and Parrinello–Rahman barostat, following the CHARMM-GUI protocol (27). A 1.5 μs production run was performed for the UndP-only system and a 1 μs production run was performed for the systems containing UndP and PglC. The temperature of each production run was maintained at 303 K. The composition of all the membrane bilayers studied are listed in Table S3. All simulations were performed using the GROMACS 2018.3 program (34).

The liquid-crystal order parameter (P_2_) was computed for lipids using the angle (θ) between the director vector defined by a subset of phospholipid atoms and the bilayer normal vector where:

P_2_ = 0.5(3 < cos ^2^(θ) > - 1)

For POPE and POPG, the director vectors were defined by the C1 through C16 carbon atoms and C1 through C14 carbon atoms. For CL, the director vectors were defined by the C1 through C12 carbon atoms (Fig. S1).

The 2D bond-orientational order parameter ($\Psi_{6}^{k}$) was calculated by considering one atomic coordinate per lipid chain using:

$${|\Psi }_{6}^{k}|=|\frac{1}{6}\sum_{l=1}^{6}e^{i6\theta_{kl}}|$$

where $\theta_{kl}$ is the angle between an arbitrary vector and the vector connecting the central atom (k) and one of the six nearest-neighbor atoms (l). Voronoi tessellation was also performed considering the position of the lipid chains. For POPE and POPG, the coordinates of the C14 and C16 carbon atoms, and for CL, the coordinates of the C12 carbon atoms, were used to define the positions of the lipid chains (Fig. S1). The electrostatic potential maps were calculated using PDB2PQR (35), Adaptive Poisson-Boltzmann Solver (36), and APBSmem (37). The relative hydrophobicity or hydrophilicity of the model surface was mapped using the Eisenberg hydrophobicity scale (38). Principal component analysis for the protein was performed by diagonalizing the covariance matrix of the position of the backbone atoms using ProDy (39) and GROMACS tools. All other analyses were performed using in-house code written using the MDAnalysis Python library (40).

### Preparation of site-directed variants

Single and double point mutations were introduced into the WT *C. concisus* SUMO-PglC sequence in the pE-SUMO vector (6) using the QuikChange II Site-Directed Mutagenesis kit (Agilent) and primers in Table S4.

### Protein purification

WT and SUMO-PglC variants were expressed and purified using published protocols (6). Briefly, proteins were expressed in *Escherichia coli* BL21-DE3-RIL cells by autoinduction. Cell pellets were resuspended in base buffer (50 mM Hepes pH 7.5 and 100 mM NaCl) supplemented with 25 mg lysozyme, 25 μl DNAse I, and 50 μl protease inhibitor cocktail. Cells were sonicated twice for 1.5 min (1 s on/2 s off, 50% amplitude), and the lysed cells were centrifuged at 9417*g* for 45 min. The resulting supernatant was centrifuged at 142,414*g* for 65 min to pellet the cell envelope fraction. The cell envelope fraction was homogenized into base buffer with 1% n-dodecyl-β-D-maltoside (DDM) and rotated overnight at 4 °C. The detergent-homogenized sample was centrifuged at 161,571*g* for 65 min. The supernatant was incubated with 1 ml Ni-NTA resin for 1 h at 4 °C. The resin was washed with 20 column volumes of Wash I Buffer (base buffer, 0.03% DDM, 20 mM imidazole, and 5% glycerol), followed by 20 column volumes Wash II Buffer (base buffer, 0.03% DDM, 45 mM imidazole, and 5% glycerol). SUMO-PglC was eluted in two column volumes of elution buffer (base buffer, 0.03% DDM, 500 mM imidazole, and 5% glycerol) and immediately desalted using a 5 ml HiTrap Desalting Column into 50 mM Hepes pH 7.5, 100 mM NaCl, 0.03% DDM, 5% glycerol. Protein purity was assessed by SDS-PAGE with Coomassie staining (Fig. S11).

### Activity assays

Assays for PGT activity were performed using the UMP-Glo assay (Promega). Reactions contained 20 μM UndP, 20 μM UDP-diNAcBac, 50 mM Hepes pH 7.5, 100 mM NaCl, 5 mM MgCl2, 0.1% Triton X-100, and 10% dimethylsulfoxide with 0.4 nM SUMO-PglC. Reactions in the linear range were quenched with the UMP-Glo detection reagent and luminescence was measured in a plate reader as previously described (8). Relative luminescence units were converted into concentration of UMP (μM) with a standard curve.

### Crystallization

WT-SeMet *C. concisus* PglC used for crystallography was prepared as described previously (6). Notably, PglC copurifies with approximately three phosphatidylethanolamine: one phosphatidyl glycerol endogenous lipid observed by thin layer chromatography. WT Se-Met PglC crystals were grown by hanging drop vapor diffusion at 17 °C from a 1:1 mix of protein and well comprised of 0.1 M Bis–Tris pH 6.0, 0 .3 M MgCl_2_, 27% PEG 3350, and 1 mM tris(2-carboxyethyl)phosphine (2 μl total volume). Se-Met PglC (276 μM in in 50 mM Hepes pH 7.5, 100 mM NaCl, and 0.03% DDM) was used after incubation with 1 mM UDP on ice for 30 min. WT-SeMet PglC crystals used for data collection appeared within 3 days and reached their final size after 14 days. Crystals were flash cooled by plunging into liquid nitrogen for transport and data collected without additional cryo-protection.

### Data collection and refinement

The WT-SeMet PglC dataset was collected at BNL NSLS-II 17-ID-1 (AMX) at the Se X-ray absorption energy peak (12,665 eV) allowed initial partial phases to be solved by single-wavelength anomalous dispersion using the Phenix suite (41). Matthews coefficient analyses for the dataset were consistent with eight copies in the asymmetric unit. Data were scaled and integrated using XDS (https://xds.mr.mpg.de) (42). SHELXD (43) was run for 5000 trials with a resolution cutoff of 4.5 A to identify 16 Se sites. PHENIX.SOLVE (https://phenix-online.org/) (44) was used to find an additional 6 Se sites and calculate subsequent Se substructure phases for 22 out of the expected 32 Se atoms in the ASU. PHENIX.RESOLVE (45) was used to perform initial solvent flattening and phase-extension. The partial model resulting from these maps was utilized, together with data from a second native I57M/I87M 2.59 Å dataset to determine the published structure of the more complete, higher I/σ(I) dataset of I57M/Q175M PglC at 2.74 Å resolution (PDB 5W7L) (6). This higher resolution model was ultimately used to calculate the phases for the WT-SeMet PglC dataset.

Refinement against the electron density map was performed with PHENIX.Refine (46) to refine XYZ coordinates, real-space, rigid body, and group B-factors. Subsequent rounds of refinement included refinement of translation libration-screw parameters, manually placed waters, and simulated annealing of Cartesian coordinates and torsion angles. The final model with eight subunits in the ASU was refined to R_work_/R_free_ of 0.27/0.30 with no significant outliers using PHENIX.Refine (46). All chains of the model contain 185 out of 205 amino acids as density corresponding to the C terminus was not visible. Data collection and refinement statistics are tabulated in Table S2. The coordinates were deposited in the PDB under entry 8E37.

### Electron density analysis

The mFo-DFc map of WT-SeMet *C. concisus* PglC in CCP4 format was generated using PHENIX (41) from an MTZ file containing map coefficients. The total electron density in each active site of PglC protomers was calculated using MAPMAN (47). The peaks in the mFo-DFc map at 3.0 σ were identified and the electron density in a 3.5 Å sphere surrounding peaks was integrated and the integrals were added as previously described (48) to determine the total electron density in the volume where UDP is expected to bind.

## Data availability

All data generated or analyzed during this study are included in this article. The structure factors and coordinates were deposited in the Protein Data Bank under accession code 8E37.

## Supporting information

This article contains supporting information.

Table S1 (7) Activities of PglC Variants (Table S2); data collection and refinement statistics (Table S3); membrane composition studied (Table S4); mutagenesis primers (Fig. S1); lipid structures (Fig. S2); histograms of order parameters (Fig. S3); lateral lipid organization (Fig. S4); area of lipids in the absence of CL (Fig. S5); area of lipids and radius of gyration of UndP (Fig. S6); insertion depth of PglC (Fig. S7); comparison of RMSF and B-factor (Fig. S8); position of basic residues in WT and variant form of PglC (Fig. S9); overlay of WT and variant forms of PglC (Fig. S10); NanoDSF data for PglC (Fig. S11); and SDS-PAGE analysis.

## Conflict of interest

The authors declare that they have no conflicts of interest with the contents of this article.
