# Supplementary material for: Synergistic computational and experimental studies of a phosphoglycosyl transferase membrane/ligand ensemble
Source: J Biol Chem. 2023 Aug 25;299(10):105194. doi: 10.1016/j.jbc.2023.105194 (PMC10519829; doi:10.1016/j.jbc.2023.105194)
Supplement: Supporting information [file mmc1.pdf]

Supporting Information for

Synergistic computational and experimental studies of a phosphoglycosyl transferase membrane/ligand ensemble

Ayan Majumder[1], Nemanja Vuksanovic[1], Leah C. Ray[2], Hannah M. Bernstein[3], Karen N. Allen[1,2,*], Barbara Imperiali[3,4,*], John E. Straub[1,*]

[1]Department of Chemistry, Boston University, 590 Commonwealth Ave, Boston, MA 02215, USA

[2]Program in Biomolecular Pharmacology, Boston University School of Medicine, Boston, MA 02118, USA

[3]Department of Biology, Massachusetts Institute of Technology, 31 Ames St, Cambridge, MA 02139, USA

[4]Department of Chemistry, Massachusetts Institute of Technology, 77 Massachusetts Ave, Cambridge, MA 02139, USA

*Correspondence should be addressed to Karen N. Allen, drkallen@bu.edu; Barbara Imperiali, imper@mit.edu; John E. Straub, straub@bu.edu

Table S1. Summary of Activities of PglC Variants

entry[a]	mutation	activity[b]
1	WT	+++
2[c]	R88Q	-
3	R145A	+
4	R145Q	+
5	K179A	+++
6	K179Q	+++
7	K182A	+++
8	K182Q	+++
9	K179A/K182A	+++
10	K179Q/K182Q	+++

[a]Assays were performed in duplicate under identical conditions with WT or SUMO-PglC variants with the substrate concentrations set at the K_m values: 20 μM UDP-diNAcBac and 20 μM UndP. All activity measurements are in the linear response range. [b]The wild-type level of activity with 0.4 nM enzyme, to which all mutant enzymes are compared, is defined as +++. A designation of +++ would represent the activity of an enzyme that attains >50-75% of wild-type activity with 0.4 nM enzyme. A designation of ++ would represent the activity of a mutant enzyme that attains ~75% of wild-type activity with 4 nM (10-fold wild-type concentration) enzyme. A designation of + would represent the activity of a mutant enzyme that attains ~75% of wild-type activity with 40 nM (100-fold wild-type concentration) enzyme. A designation of – is used to describe the activity of a mutant enzyme that attains <30% of wild-type activity with 100-fold wild-type enzyme concentration. [c] published in [Biochemistry. 2015;54(50):7326-34.]

Table S2. Data collection and refinement statistics

	WT SeMet PglC PDB ID 8E37
**Data collection**	
Beamline	BNL NSLS-II 17-ID-1 (AMX)
Wavelength (Å)	1.0<br>0
Resolution range (Å)	47.737 – 3.013 (3.121- 3.01)
Space group	P 31 2 1
Unit Cell (Å)	a = b = 142.82,<br>c = 192.563
Total Reflections	75337
Unique reflections	38779 (3544)
Multiplicity	1.9 (2.0)
Completeness (%)	82.76 (76.47)
Mean I/σ(I)	14.2 (1.0)
Wilson B-factor	83.8
R_{merge}	0.074 (0.656)
R_{meas}	0.105 (0.928)
$CC_{1/2}$	0.98 (0.553)
**Refinement**	
Reflections used in refinement	38391 (3428)
Reflections used for R_{free}	1994 (183)
R_{work}	0.2657 (0.3539)
R_{free}	0.2959 (0.3532)
Number of non-hydrogen atoms	12088
Protein residues	1480
RMS(bonds)	0.002
RMS(angles)	0.61
Ramachandran favored (%)	95.90
Ramachandran outliers (%)	0.34
Rotomer outliers (%)	0.00
Clashscore	7.73
Average B-factor	111.53
Number of TLS groups	8

Table S3: Membrane compositions simulated in this study

# of membrane components	Total simulation
148 POPE + 52 POPG	1 μs
134 POPE + 46 POPG + 20 CL	1.5 μs
2 UndP + 134 POPE + 46 POPG + 20 CL	1.5 μs
1 PglC + 268 POPE + 92 POPG + 40 CL	600 ns
1 PglC + 2 UndP + 268 POPE + 92 POPG + 40 CL	1 μs
1 mutant PglC + 2 UndP + 268 POPE + 92 POPG + 40 CL	1 μs

Table S4: ***C. concisus*** **PglC variant site-directed mutagenesis primers**

construct	primer	
R145A	primer #1	CGCAGGTAAATGGCGCAAACGCCATAAGTTGGG
	primer #2	CCCAACTTATGGCGTTTGCGCCATTTACCTGCG
R145Q	primer #1	CGCAGGTAAATGGCCAAAACGCCATAAGTTGGGAG
	primer #2	CTCCCAACTTATGGCGTTTTGGCCATTTACCTGCG
K179A	primer #1	GCCTTACAGACAATAGAAGCGGTGCTAAAACGAAGTGG
	primer #2	CCACTTCGTTTTAGCACCGCTTCTATTGTCTGTAAGGC
K179Q	primer #1	GCCTTACAGACAATAGAACAGGTGCTAAAACGAAGTG
	primer #2	CACTTCGTTTTAGCACCTGTTCTATTGTCTGTAAGGC
K182A	primer #1	CAATAGAAAAGGTGCTAGCACGAAGTGGTGTCAGCAAAG
	primer #2	CTTTGCTGACACCACTTCGTGCTAGCACCTTTTCTATTG
K182Q	primer #1	CAATAGAAAAGGTGCTACAACGAAGTGGTGTCAGC
	primer #2	GCTGACACCACTTCGTTGTAGCACCTTTTCTATTG
K179A/ K182A	primer #1	CCTTACAGACAATAGAAGCGGTGCTAGCACGAAGTGGTGTCAGC
	primer #2	GCTGACACCACTTCGTGCTAGCACCGCTTCTATTGTCTGTAAGG
K179Q/ K182Q	primer #1	CTTACAGACAATAGAACAGGTGCTACAACGAAGTGGTGTC
	primer #2	GACACCACTTCGTTGTAGCACCTGTTCTATTGTCTGTAAG

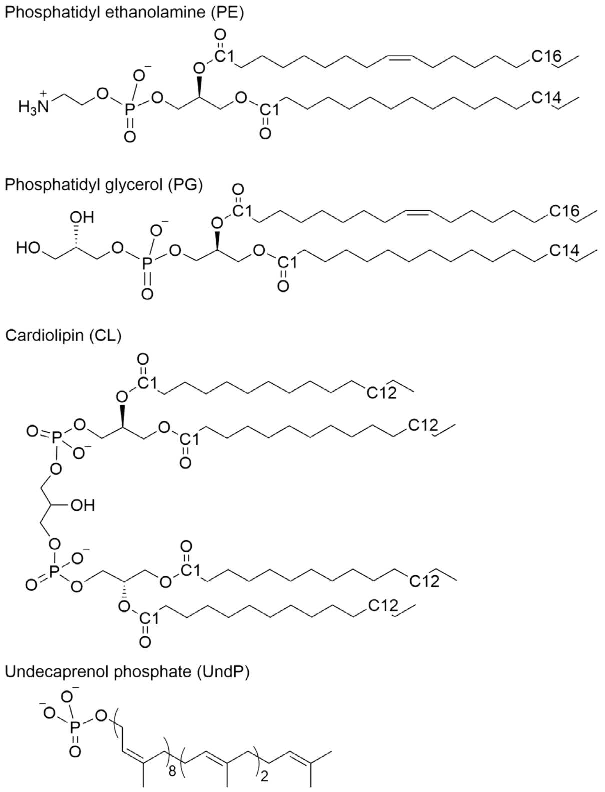

Figure S1: Structures of lipids included in the model bacterial membrane used in the simulation. The most common acyl chain modifications were used for each lipid. C1, C12, C14, and C16 atom positions of the lipids obtained from the trajectory were used for analysis.

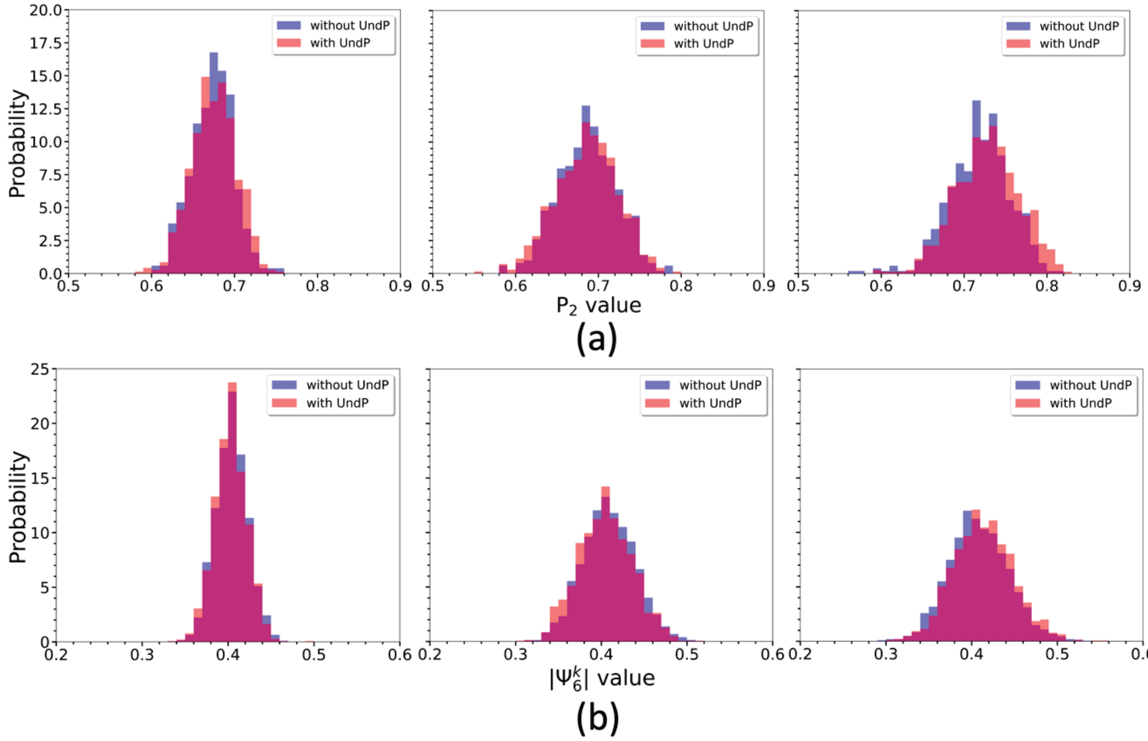

Figure S2: Probability distribution of P_2 and $|\Psi_6^k|$ order parameters of different membrane components obtained from simulation of membrane with and without the inclusion of UndP.

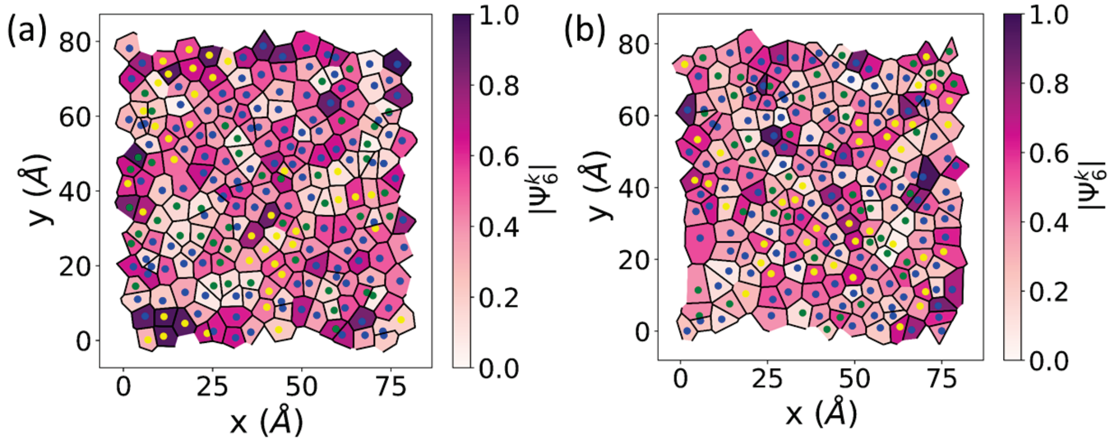

Figure S3: Voronoi tessellation showing lateral organization of lipids in the (a) upper and (b) lower leaflets in the final frame of the simulation. POPE, POPG, and CL are represented by blue, green, and yellow dots in center, respectively. Color of cell represents the value of the $|\Psi_6^k|$ order parameter providing a measure of local hexagonal packing.

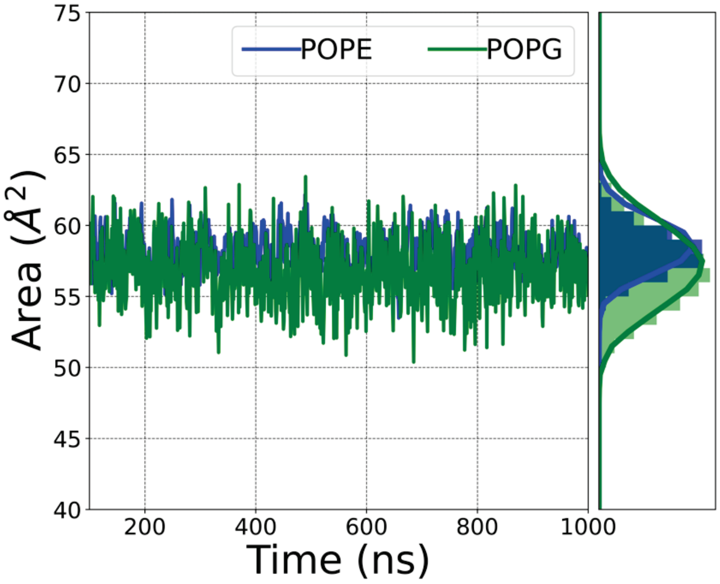

Figure S4: Instantaneous area of lipid components obtained by performing a Voronoi tessellation shown as a time series over the course of the simulation as an aggregate distribution for POPE (blue), and POPG (green). The simulation was performed with a membrane bilayer of composition 74 mol% POPE and 26 mol% POPG (no CL).

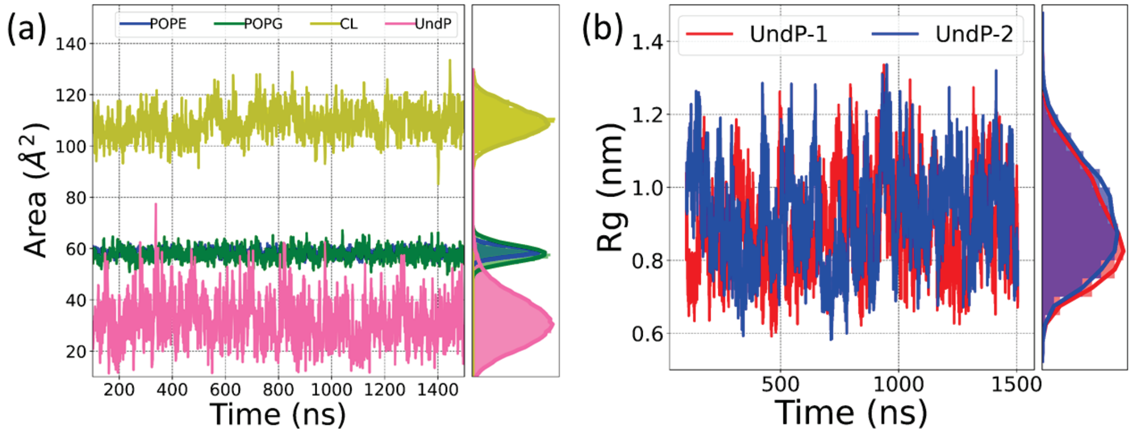

Figure S5: (a) Instantaneous area of lipid components obtained by performing a Voronoi tessellation shown as a time series over the course of the simulation an as aggregate distribution for POPE (blue), POPG (green), CL (yellow), and UndP (mauve). (b) Radius of gyration of UndP molecules shown as time series and aggregate distribution.

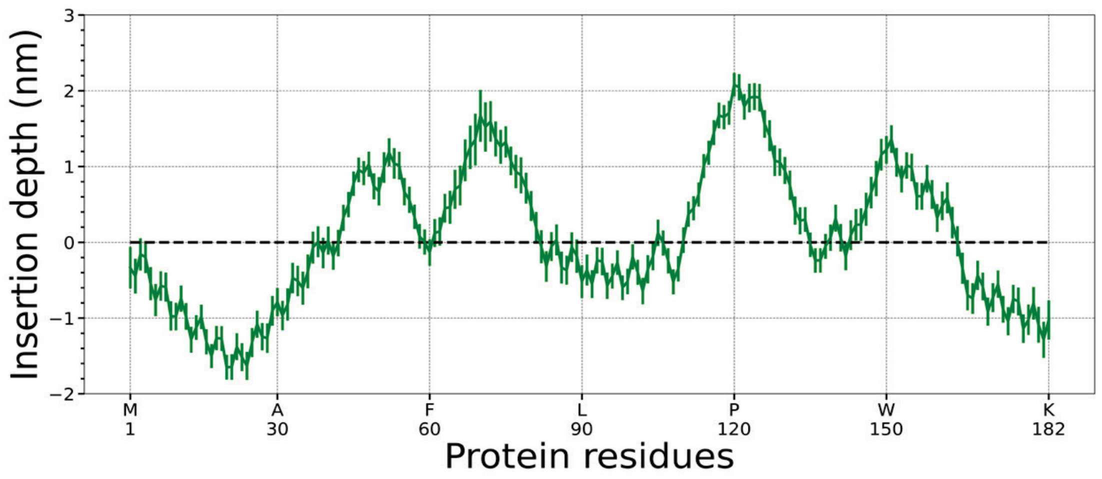

Figure S6: Average depth of insertion of PglC residues in the membrane bilayer averaged over the simulation. Oscillations indicate presence of helices. Error bars represent one standard deviation in the distribution of insertion depth.

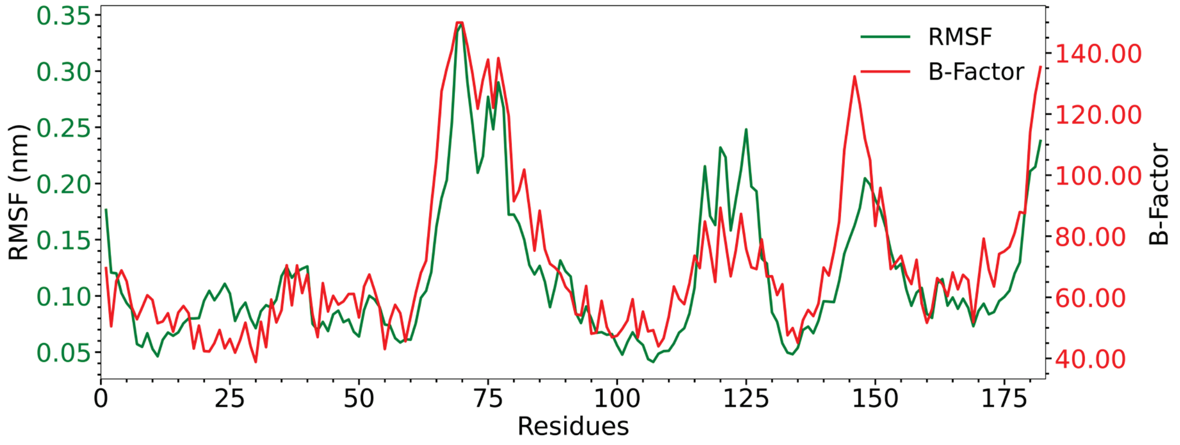

Figure S7: RMSF of PglC structures obtained from simulation and B-factor values obtained from the experiments.

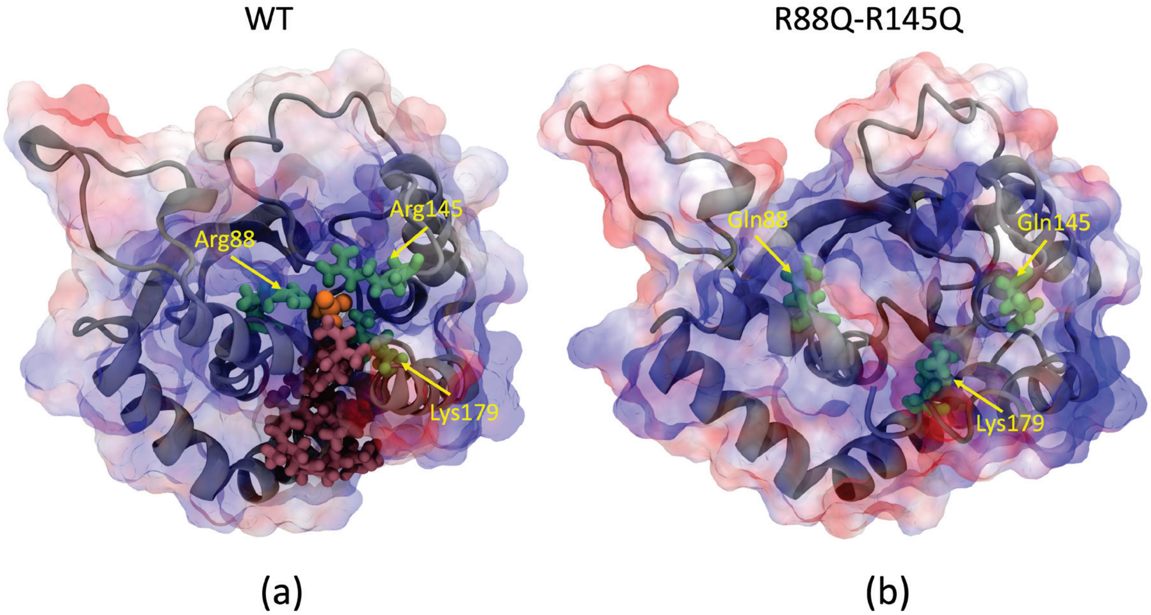

Figure S8: Ribbon diagram with overlayed electrostatic potential surface of (a) WT and (b) R88Q-R145Q PglC showing difference in the proximity of basic residues near the active site.

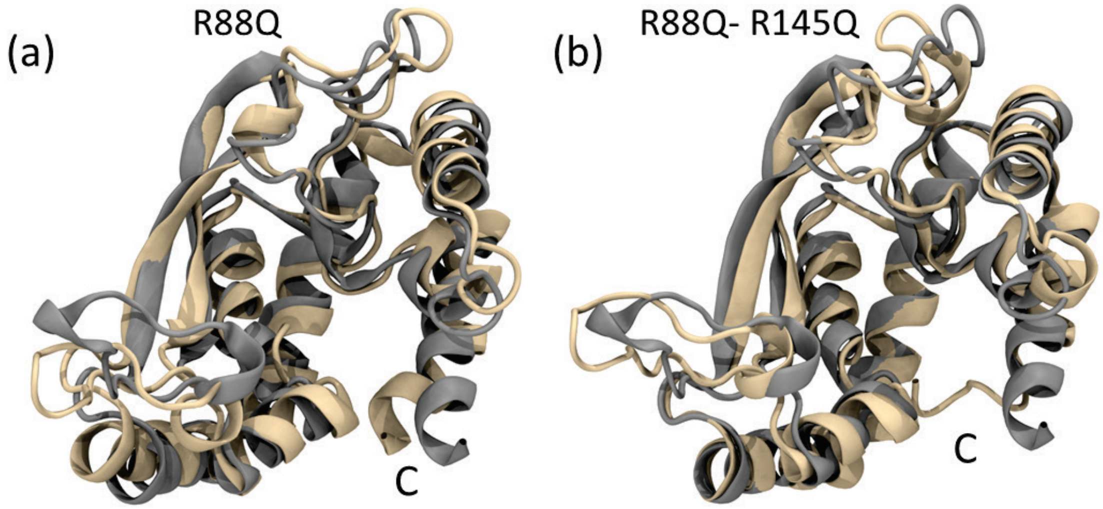

Figure S9: Overlay of representative instantaneous structures of (a) WT (gray) and R88Q PglC (wheat) and (b) WT (gray) and R88Q-R145Q PglC (wheat). The C-terminus of the PglC is marked by “C”.

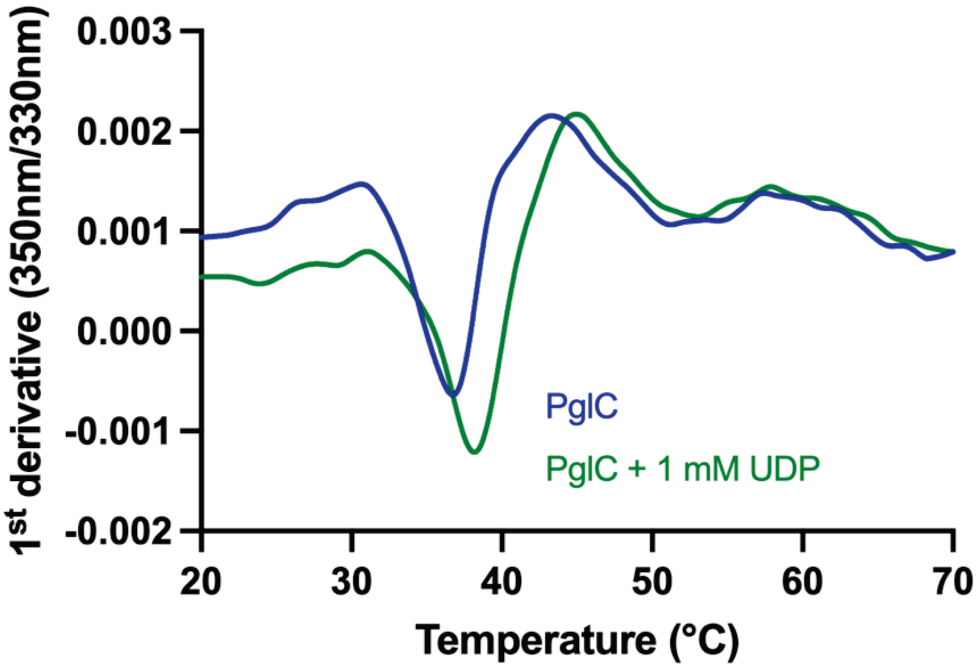

Figure S10: Plot representing the mean values of the first derivative of the 350/330 nm tryptophan emission of recombinant *C. concisus* PglC, demonstrating an increase in thermal stability upon addition of UDP. The presence of UDP increases the onset of melting by 1.9 ± 0.3°C. Triplicate incubations of 303 μM PglC and 1 mM UDP were carried out on ice for 30 minutes in 50 mM HEPES pH 7.5, 150 mM NaCl, 0.03% DDM, 5% glycerol, 5 mM $MgCl_2$, 1 mM DTT. The nanoDSF readings were performed on a Prometheus NT.48 using 20% excitation power and heating slope of 1 °C/min.

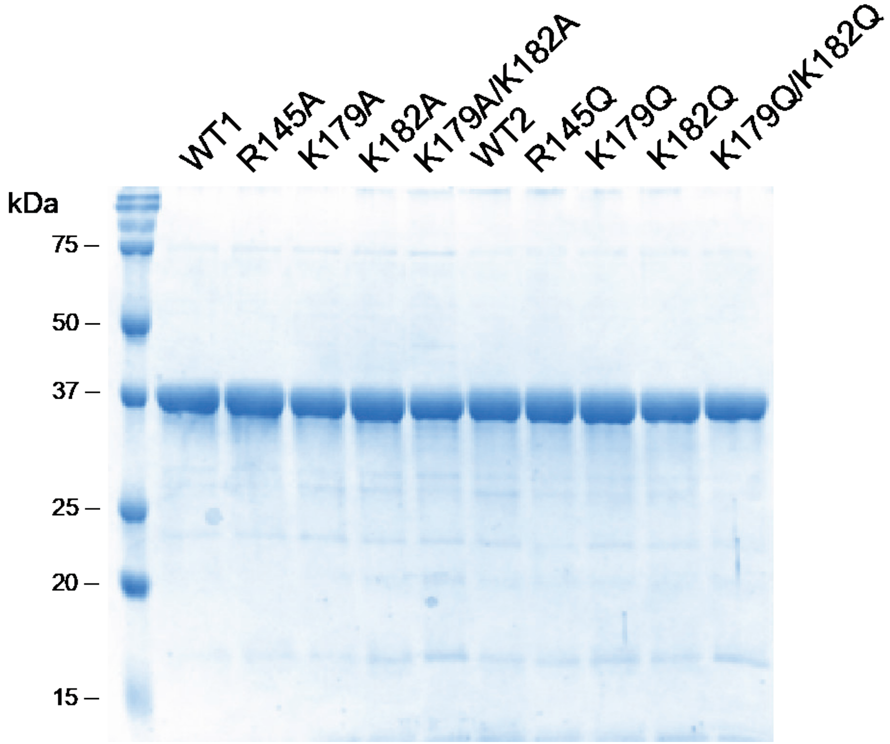

Figure S11: SDS-PAGE analysis of purified His$_6$-SUMO-PglC proteins. His$_6$-SUMO-PglC WT (technical duplicates WT1 and WT2) and variants made by site-directed mutagenesis (label across top of gel) were obtained employing Ni-NTA affinity chromatography. The expected molecular weight of the tagged proteins is 35.8 kDa.
